# Isolated Upper Extremity Posttransplant Lymphoproliferative Disorder in a Child

**DOI:** 10.1155/2015/813989

**Published:** 2015-06-18

**Authors:** Sarah E. Halula, Daniel G. Leino, Manish N. Patel, John M. Racadio, Matthew P. Lungren

**Affiliations:** ^1^Division of Pediatric Interventional Radiology, Department of Radiology, Cincinnati Children's Hospital Medical Center, 3333 Burnet Avenue, Cincinnati, OH 45229-3030, USA; ^2^Division of Pathology, Cincinnati Children's Hospital Medical Center, 3333 Burnet Avenue, Cincinnati, OH 45229-3039, USA; ^3^Department of Radiology, Stanford University Medical Center, Lucile Packard Children's Hospital, 725 Welch Road, Room 1675, Stanford, CA 94305-5913, USA

## Abstract

Posttransplant lymphoproliferative disorder (PTLD) is a well-described complication of solid organ and bone marrow transplants. The most common presentation is intra-abdominal lymphadenopathy or single or multiple intraparenchymal masses involving the liver, spleen, or kidneys. Here we describe the imaging and pathology findings of an unusual case of PTLD appearing as an intramuscular forearm lesion in a pediatric male. The manifestation of PTLD as an isolated upper extremity mass in a pediatric patient has to our knowledge not been described.

## 1. Introduction

Posttransplant lymphoproliferative disorder (PTLD) is a complication seen in nearly 1.2–30% of patients receiving solid organ or bone marrow transplant [1]. A higher incidence of PTLD has been found in children compared to adults; however, a better prognosis is demonstrated in the pediatric population [1, 2]. Of the pediatric cases of PTLD in literature, Epstein-Barr Virus is implicated in nearly 80–90%, and more than 90% are EBV-positive in early diagnoses of PTLD [1, 3].

In most cases, pediatric patients are seronegative for EBV at the time of transplantation. Subsequent development of a primary EBV infection occurs due to immunosuppressive therapy, which allows EBV-infected B-cells to undergo unimpeded proliferation. The highest incidence of PTLD is seen in small bowel transplant and heart-lung transplant, while the lowest incidence is seen in kidney and liver transplant [4]. This case describes an unusual presentation of PTLD as a localized forearm lesion in a 3-year-old male after multiorgan transplantation.

## 2. Case Presentation

A 3-year-old male underwent liver and intestinal transplant in January 2013 for Hirschsprung disease. Past medical history also included small bowel graft versus host disease. Pertinent medications included tacrolimus (Prograf) 1 mG capsule 2 mg 2x daily. In November 2013, 10 months after the multivisceral organ transplant, the patient developed swelling in the right forearm.

**Figure 1:**
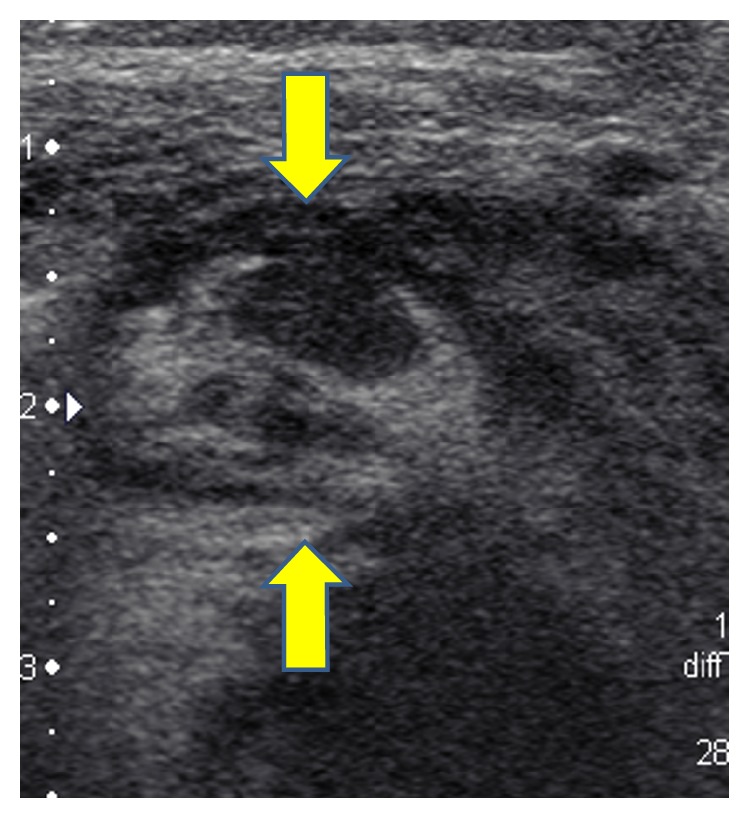
Selected gray-scale axial ultrasound imaging of the right forearm soft tissue mass. A heterogeneous echogenic mass is demonstrated along the ventral forearm musculature displacing, rather than invading, surrounding structures (arrows).

**Figure 2:**
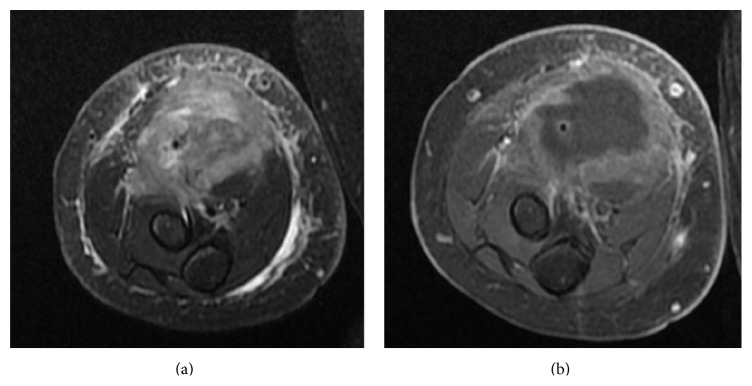
(a) Fat-saturated T2 weighted axial image of the right forearm mass demonstrates a hyperintense lesion within the anterior compartment with edema within the adjacent subcutaneous fat and muscle. (b) Contrast enhanced fat-saturated T1 weighted images of the same level demonstrate irregular peripheral enhancement of the lesion.

**Figure 3:**
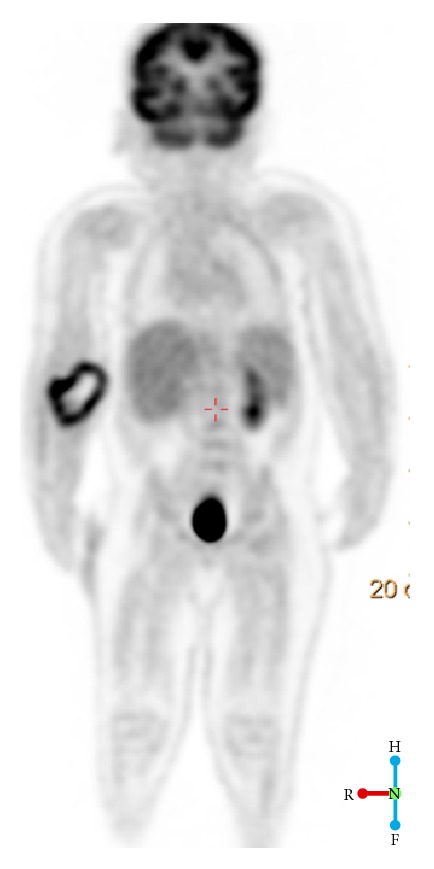
Whole body FDG PET imaging reveals a FDG-avid mass in the right forearm with central photopenia corresponding to the mass within the ventral aspect of the proximal forearm.

Initial ultrasound of the palpable abnormal area demonstrated an ill-defined heterogeneous deep soft tissue mass in the anterior forearm musculature (Figure 1). No significant internal vascularity by Color Doppler was observed. The ultrasound was concerning for an inflammatory process or subacute hematoma. An MRI was performed which demonstrated a 7.2 × 4.1 × 2.9 cm (CC × TR × AP) heterogeneous lesion—predominantly iso- to hypointense T1 and mildly hyperintense T2—in the anterior soft tissue of the right forearm (Figure 2). The lesion demonstrated peripheral rim enhancement. Adjacent edema within the subcutaneous fat and muscles was present, along with fluid signal along the superficial fascial planes of the forearm musculature. Subsequently, a whole body FDG PET-CT revealed a large focus of peripheral increased FDG uptake along the ventral aspect of the proximal forearm extending into the antecubital fossa (Figure 3). The differential diagnosis included myositis ossificans, infection, and hematoma with secondary infection. PTLD was not initially raised due to the location and size of the mass.

**Figure 4:**
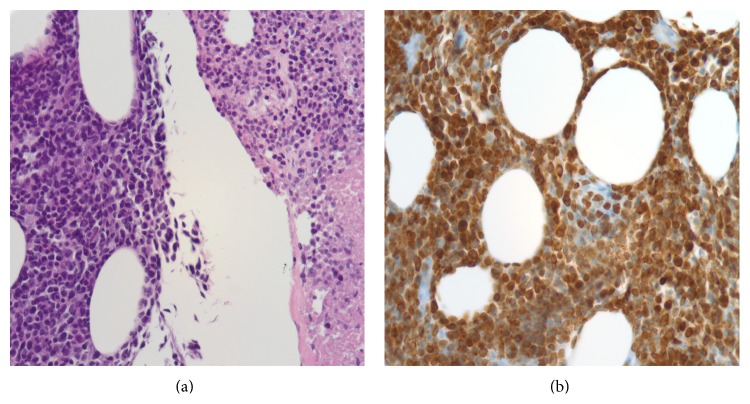
Histomorphologic and immunohistochemical analysis. (a) Relatively monotonous population of atypical lymphocytes infiltrating in adipose tissue with round to angulated nuclei, condensed nuclear chromatin, and inconspicuous nucleoli. The right side shows the same population of cells from the left in various stages of necrosis (H&E stained, formalin fixed, paraffin embedded section, at 40x magnification). (b) Immunohistochemical stain for CD79a, a B-cell specific antigen (formalin fixed, paraffin embedded section, at 40x magnification).

Due to the atypical presentation and the lack of a trauma history or supporting clinical evidence for infection, ultrasound guided biopsy of the mass was performed. Histomorphologic analysis revealed an atypical, diffuse lymphoid infiltrate composed of a relatively monotonous population of cells. By immunohistochemical staining, these cells were diffusely positive for CD79a (Figure 4), a subset immunoreactive to CD20, and all were negative for CD3, all indicative of a B-cell lineage. CD30 was present on many cells of variable size including some larger forms with a membranous and perinuclear dot-like pattern. The MIB-1 (Ki-67) showed a proliferation index of approximately 70%. In situ hybridization with EBER (EBV) showed strong positivity (nuclear) in several clusters of the atypical B-cells. PCR viral studies performed on the tissue confirmed a diagnosis compatible with posttransplant lymphoproliferative disorder (PTLD), monomorphic type [5].

The patient's treatment regimen was reduced to tacrolimus (1 mg) with daily check of tacrolimus blood levels. Following this change, the patient's imaging studies showed further worsening of his disease. Cyclophosphamide (500 mg/m^2^/day) was started per Children's Oncology Group protocol ANHL002, which also included doxorubicin 60 mg/m^2^, brentuximab (1.8 mg/kg IV), ofatumumab (600 mg/m^2^ IV), and filgrastim (5 mcg/kg IV daily). Despite efforts from the health care team, the patient developed overwhelming viremia with EBV, thought to be secondary to tumor lysis syndrome. The patient died of respiratory arrest secondary to septic shock approximately 3 months after presenting with the forearm lesion.

## 3. Discussion

PTLD is a potentially devastating disease that has been described to occur throughout the chest and abdomen, the central nervous system, and rarely the extracranial head and neck [3]. The overall prevalence of EBV-associated PTLD following solid organ transplant (SOT) ranges from 1% to 20%, with rates varying according to the type of organ transplanted, pretransplant EBV serostatus, and the age of the recipient [3, 4, 6, 7]. The clinical presentation is variable, both in symptomatology and in severity, ranging from flu-like symptoms with fever and malaise to fulminant systemic disease [6]. EBV-positive PTLD typically presents relatively early after transplant with the highest incidence occurring in the first year after transplant—although later cases do occur [4, 6, 7]. As a general rule, patients who present late (>1 year) have more aggressive tumors and a poor prognosis [6–9].

In children, the distribution of disease is as variable as the adult transplant population. Although it is commonly held that PTLD most often occurs in the anatomic region of solid organ transplant in pediatric recipients, there is evidence that even patients with early PTLD show extralymphatic (e.g., gastrointestinal) manifestation unrelated to the grafted organ. Furthermore, PTLD in later stages is often nodal in nature and unrestrained to the anatomic region of transplant. The literature does suggest that the highest prevalence of renal-based PTLD is in kidney transplant recipients, while lung-based and liver-based PTLD arises preferentially in recipients of those organs [10]. Adenotonsillar forms of PTLD are more commonly seen in pediatric transplant patients; less common sites of PTLD include the extracranial head and neck, including the superficial ocular region. The imaging appearance of musculoskeletal PTLD, such as that which presented in the patient's upper extremity, has not been previously described in the pediatric population in the literature.

Often, the key to making the diagnosis is identification of lymphadenopathy or solid mass anywhere in the body of an organ transplant patient and maintaining a high index of suspicion in the transplant patient population should any new masses or lesions arise [3]. PTLD imaging workup includes body CT, MR, and/or PET-CT to identify asymptomatic lesions when PTLD is suspected or when PTLD is diagnosed to allow for staging [6, 11–13]. In general, extranodal involvement is 3-4 times more common than nodal involvement and resembles primary lymphoma of those organs. Typically extranodal masses are hypodense on CT with variable enhancement. The MR appearance is typically hypointense on T1WI and iso- to slightly hyperintense on T2WI and often shows subtle peripheral enhancement, which was consistent with our case. Typical appearance of a PTLD mass lesion includes increased FDG uptake and can be seen with internal necrosis.

Ultimately, biopsy of lesions or sites of disease is needed to definitively diagnose PTLD and rule out other opportunistic infections that might require alternate therapy or be present concurrently [9]. In a majority of cases, biopsy reveals monomorphic, monoclonal B-cell positive for EBV, as in our case. The positive EBV cytology was expected as the patient's therapy included immunosuppressive tacrolimus. As is suggested in the literature, the patient's treatment for PTLD included reduced immunosuppression balanced with risk of transplant rejection [14]. Current guidelines for the treatment of PTLD, however, include reduction of immunosuppression, as well as treatment with anti-CD20-antibody with or without moderate chemotherapy [3, 14]. In addition, intensive chemotherapy and experimental EBV-directed T-cell therapy or other monoclonal antibodies have been used.

Posttransplant lymphoproliferative disorder (PTLD) is a deadly disease that affects transplant patients and can manifest as a soft tissue mass anywhere in the body. PTLD should always be considered in the differential diagnosis for a soft tissue mass anywhere in the body of a transplant patient.
